# De Novo Cytomegalovirus Colitis in a Donor-Seronegative/Recipient-Seronegative Kidney Transplant Recipient

**DOI:** 10.7759/cureus.43509

**Published:** 2023-08-15

**Authors:** Zoheb I Sulaiman, Maithri P Reddy, Hasan Samra, Gina Askar

**Affiliations:** 1 Infectious Diseases, Augusta University Medical College of Georgia, Augusta, USA; 2 Internal Medicine, Dwight D. Eisenhower Army Medical Center, Augusta, USA; 3 Pathology, Augusta University Medical College of Georgia, Augusta, USA

**Keywords:** cytomegalovirus (cmv), infectious disease medicine, transplant infectious disease, invasive cmv disease, kidney transplant recipients, cmv colitis

## Abstract

Cytomegalovirus (CMV) is one of the most frequent microbes linked with kidney transplant recipients. CMV infection is typically classified as CMV virus isolation in any body fluid or specimen. We present a 43-year-old man who underwent a deceased donor kidney transplant with CMV donor-seronegative and recipient-seronegative (CMV D-/R-) status and completed three months of CMV prophylaxis with high-dose acyclovir given his low-risk status. He was admitted for complaints of profuse watery diarrhea and persistent fevers lasting one week in duration. His infectious workup led to a CMV quantitative nucleic acid amplification test (QNAT) polymerase chain reaction (PCR) of 239,977 IU/mL with a biopsy-proven diagnosis of invasive CMV colitis. He was treated inpatient with intravenous ganciclovir for two weeks and then de-escalated to oral valganciclovir until achieving viremia resolution with undetectable CMV QNAT PCR as an outpatient. This case illustrates the importance of the changing epidemiology and clinical presentation of CMV disease in solid organ transplant (SOT) recipients in an era of new immunosuppression regimens and improved CMV disease detection in the early post-transplant period.

## Introduction

Cytomegalovirus (CMV) is a significant cause of opportunistic infections in patients with solid organ transplantation (SOT). It is a ubiquitous herpes virus that infects up to 60%-100% of people in adulthood, can affect multiple organs, and present as a viral syndrome, pneumonitis, colitis, hepatitis, myocarditis, rejection, and graft dysfunction [1-7]. Usually a benign and self-limited syndrome in immunocompetent adults, CMV can lead to severe disease in immunocompromised patients including transplant recipients and AIDS patients [1,4,6,7].

Post-transplant CMV viremia manifests as reactivation of latent infection, due to transmission from the transplanted organ, or after a primary infection in seronegative individuals. This condition occurs between the first 30 and 90 days of transplantation, and individuals highest at risk are those with a CMV donor-seropositive and recipient-seronegative (D+/R-) status [1,4,6,8]. In these patients, serology has no role in diagnosing active CMV disease; therefore, histopathology from the involved tissue and/or bronchoalveolar lavage is necessary to confirm invasive disease [1].

In SOT patients, the quantitative nucleic acid amplification test (QNAT) is the preferred laboratory assay used for diagnosis, surveillance, and monitoring of CMV disease. The limitation of this test is the absence of a universally applicable viral load threshold among all transplant centers. CMV QNAT polymerase chain reaction (PCR) assays are typically calibrated using the WHO International Reference Standard. Some institutions recognize 100 copies/mL as the level of detection of the CMV viral load, while others use 300 copies/mL as their standard. Transplant infectious disease physicians consider CMV viremia without tissue invasive disease as above 2,000 IU/mL. However, the gold standard to diagnose invasive CMV disease is through tissue biopsy or histopathology.

## Case presentation

A 43-year-old male with a past medical history of end-stage renal disease and diabetes mellitus type 2 underwent a right kidney transplant in July 2022 from a cadaveric donor (CMV D-/R- status). He initiated transplant immunosuppression with tacrolimus, mycophenolate, and prednisone therapy. He also completed three months of high-dose acyclovir therapy for CMV prophylaxis in October 2022.

The patient presented six months after the transplant in January 2023 with a complaint of acute, non-bloody diarrhea and subjective fevers for one-week duration. He reported eating a burger from a local fast food restaurant and developed weakness followed by more than five watery, non-mucoid stools every day. On admission, he was febrile up to 38.9°C and labs showed an elevated creatinine of 4.17 mg/dL (baseline creatinine: 1.8-2 mg/dL) (Table 1). Infectious disease was consulted due to acute diarrhea complicated by acute kidney injury and hypovolemia with persistent fever. He tested negative for *Clostridium difficile*,* *and his PCR gastrointestinal pathogen panel was negative for *Campylobacter*, *Shigella*, *Salmonella*, pathogenic *Escherichia* *coli*, *Cryptosporidium*, *Cyclospora*, *Entamoeba histolytica*, *Giardia*, Adenovirus, Norovirus, and Rotavirus. He underwent further testing for opportunistic infections with stool ova and parasite, stool acid-fast bacilli (AFB) culture, CMV and human polyomavirus 1 (BK) virus QNAT PCR, and upper and lower endoscopy with tissue biopsy for histopathology. On hospital day two, he was started on intravenous ceftriaxone and metronidazole for empiric infectious colitis treatment.

**Table 1:** Laboratory parameters. QNAT: quantitative nucleic acid amplification test.

Laboratory Parameter	Levels at Presentation	Normal Range
Creatinine	4.17 mg/dL	0.60-1.60 mg/dL
CMV QNAT PCR	239,977 IU/mL	<54 IU/mL

**Figure 1:**
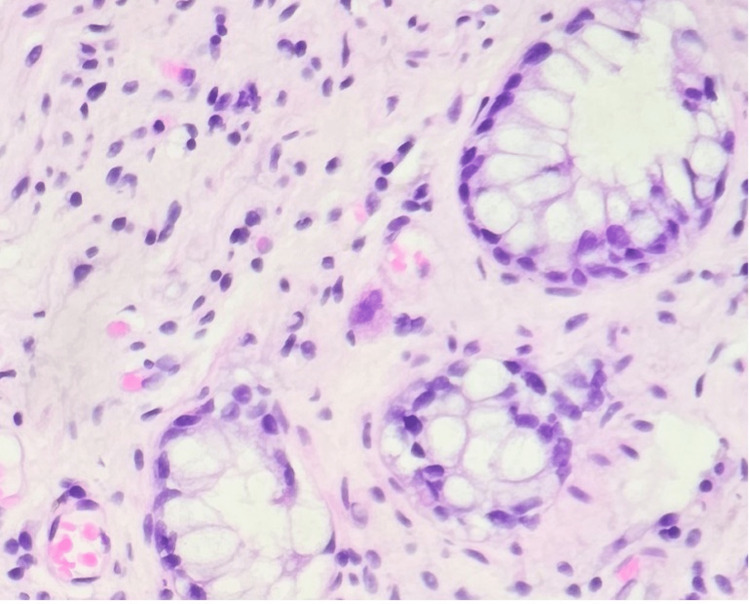
Hematoxylin and eosin section from colon tissue showing large ovoid nucleus with basophilic intranuclear inclusions.

**Figure 2:**
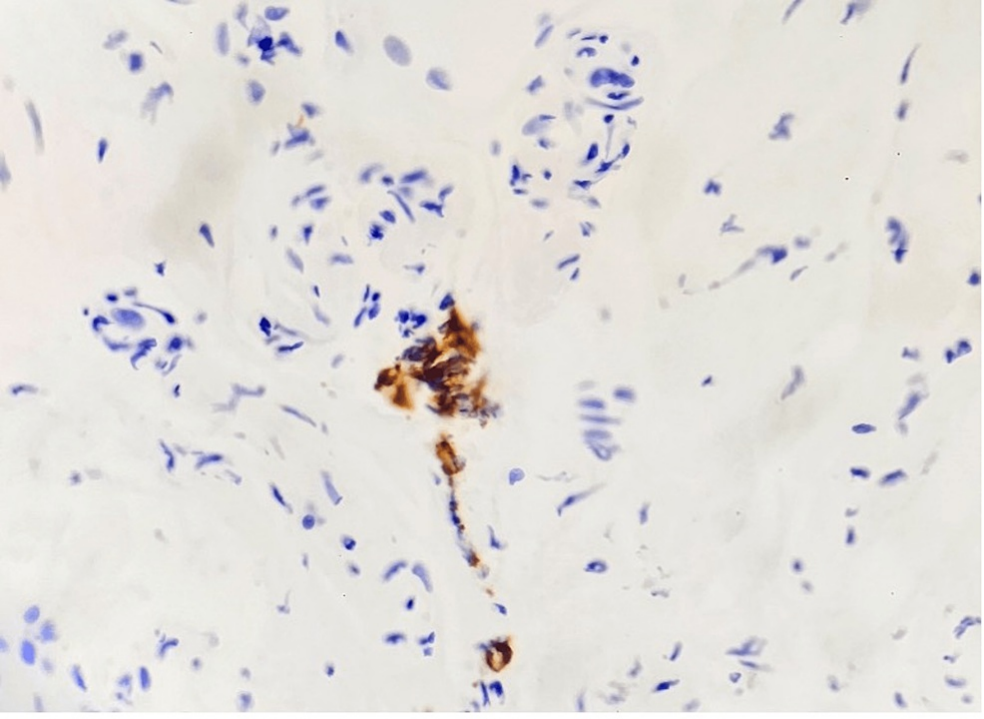
Low-power view of CMV-infected cells showing immunoreactivity with CMV immunostain. CMV: cytomegalovirus.

On hospital day five, CMV QNAT PCR returned positive at 239,977 IU/mL and he was started on renally dosed intravenous ganciclovir pending histopathology results. Antibiotics were stopped, and Epstein-Barr virus (EBV) QNAT PCR came back negative. Esophagogastroduodenoscopy showed mild gastritis with stomach biopsy negative for *Helicobacter* *pylori* and duodenal biopsy without histopathological change. Flexible sigmoidoscopy demonstrated a normal rectum and sigmoid colon. Sigmoid colon tissue biopsy revealed colonic mucosa with focal CMV viral cytopathic effect like changes. Immunohistochemical stain was negative for AFB and positive for CMV confirming a diagnosis of invasive CMV colitis (seen in Figures 1, 2). His diarrhea resolved after two weeks of intravenous ganciclovir, and he was discharged on oral valganciclovir with CMV QNAT PCR downtrending to 4,706 IU/mL prior to discharge. Valganciclovir was discontinued in the outpatient after three months of therapy when CMV QNAT PCR was noted below the detectable limit on two separate occasions.

## Discussion

This case highlights a rare presentation of invasive CMV disease in a kidney transplant recipient with CMV D-/R- status. Predisposing factors for post-transplant invasive CMV disease include mismatching CMV serology (D+/R-), those receiving lymphocyte-depleting antibodies, and lung and intestinal transplant recipients. In a 2013 study involving kidney and liver transplant recipients who received valganciclovir prophylaxis, the one-year incidence of CMV disease was 19.2% and 31.3%, respectively, in D+/R- group but only 2.5% and 3.2% in the D-/R- group [2,6].

Our patient was an immunocompromised adult, given his kidney transplant history, and had the lowest risk of developing CMV infection with his serology status of D-/R-. He acquired a primary CMV infection through natural transmission in the community prior to hospital admission, given that he had no evidence of CMV exposure before transplantation. The patient’s CMV QNAT PCR returned above 2,000 IU/mL suggesting ongoing viral replication in the peripheral blood. His colon tissue biopsy showed cytopathic effects consistent with CMV, which is diagnostic of an invasive CMV colitis. The preferred treatment for invasive CMV disease consists of intravenous ganciclovir 5 mg/kg every 12 hours and/or oral valganciclovir 900 mg every 12 hours for several weeks [6]. Our patient completed an initial two-week course of intravenous ganciclovir and then a prolonged course of oral valganciclovir until viremia resolution with undetectable CMV QNAT PCR.

## Conclusions

CMV disease should be suspected in all immunocompromised patients regardless of time post-transplant, even those who are six months or more post-transplant, due to the ubiquitous nature of the disease. With advancements in CMV prophylaxis and treatment, the rate of CMV disease in SOT recipients has decreased from 20% to 8%; however, the incidence of late cases occurring more than six months after transplantation has increased by 35%. Earlier prophylaxis and prompt treatment of invasive CMV disease can reduce both morbidity and mortality in transplant patients.
